# Opposing effects of negative emotion on amygdalar and hippocampal memory for items and associations

**DOI:** 10.1093/scan/nsw028

**Published:** 2016-03-12

**Authors:** James A. Bisby, Aidan J. Horner, Lone D. Hørlyck, Neil Burgess

**Affiliations:** ^1^UCL Institute of Cognitive Neuroscience, University College London, London, WC1N 3AZ; ^2^UCL Institute of Neurology, University College London, London, WC1N 3BG, UK

**Keywords:** hippocampus, amygdala, item memory, associative memory

## Abstract

Although negative emotion can strengthen memory of an event it can also result in memory disturbances, as in post-traumatic stress disorder (PTSD). We examined the effects of negative item content on amygdalar and hippocampal function in memory for the items themselves and for the associations between them. During fMRI, we examined encoding and retrieval of paired associates made up of all four combinations of neutral and negative images. At test, participants were cued with an image and, if recognised, had to retrieve the associated (target) image. The presence of negative images increased item memory but reduced associative memory. At encoding, subsequent item recognition correlated with amygdala activity, while subsequent associative memory correlated with hippocampal activity. Hippocampal activity was reduced by the presence of negative images, during encoding and correct associative retrieval. In contrast, amygdala activity increased for correctly retrieved negative images, even when cued by a neutral image. Our findings support a dual representation account, whereby negative emotion up-regulates the amygdala to strengthen item memory but down-regulates the hippocampus to weaken associative representations. These results have implications for the development and treatment of clinical disorders in which diminished associations between emotional stimuli and their context contribute to negative symptoms, as in PTSD.

## Introduction

It is generally accepted that increases in emotion can enhance memory. Emotional events are often remembered vividly and with great accuracy and confidence (Brown and Kulik, 1977; Bradley *et al.*, 1992; Cahill *et al.*, 1996; Kensinger and Corkin, 2003; Dolcos *et al.*, 2005; LaBar and Phelps, 2005; Sharot and Yonelinas, 2008). The amygdala plays a crucial role in emotional memory enhancements (Cahill *et al.*, 1996; Hamann *et al.*, 1999) and is thought to facilitate memory encoding via modulation of key memory structures in the medial temporal lobes (Cahill and McGaugh, 1998; Hamann, 2001; Dolcos *et al.*, 2005; Phelps and LeDoux, 2005; LaBar and Cabeza, 2006).

Increases in emotional arousal during an experience are thought to facilitate memory through enhanced consolidation processes (McGaugh, 2000, 2004). However, behavioural evidence demonstrates that not all aspects of memory are enhanced by emotion in a similar way. Studies have consistently shown that memory for the emotional content of an event (Cahill and McGaugh, 1995; Hamann *et al.*, 1999; Canli *et al.*, 2000) or the subjective sense of remembering attached to a memory (Sharot *et al.*, 2004; Dolcos *et al.*, 2005; Rimmele *et al.*, 2011) is enhanced. However, memory for associations between individual items or items and their context can be impaired (Anderson and Shimamura, 2005; Mather, 2007; Touryan *et al.*, 2007; Pierce and Kensinger, 2011; Madan *et al.*, 2012; Chiu *et al.*, 2013; Bisby and Burgess, 2014). It should be noted that subtle differences in experimental procedures can also affect the observed pattern of results for associations that include negative information. For example, it has been shown that a longer delay between encoding and test can sometimes result in better memory for negative associations (Pierce and Kensinger, 2011), which may relate to differences in forgetting rates across neutral and negative memories (Yonelinas and Ritchey, 2015). In addition, the way in which memory is tested can alter associative memory performance. Whether a negative item is presented to cue retrieval or is the target associate to be retrieved can also affect overall performance (Bisby and Burgess, 2014; Mickley Steinmetz *et al*., 2015). The neural mechanisms that contribute to this complex pattern of memory for emotional events remain unclear.

Memory for associations between items and between items and their context relies on mechanisms that go beyond those supporting memory for a single item. The former type of memory (i.e. relational memory, including associative and contextual processing) are thought to be dependent on the hippocampus, an essential structure for episodic memory (O’Keefe and Nadel, 1978; Cohen and Eichenbaum, 1993; Burgess *et al.*, 2002; Davachi, 2006; Diana *et al.*, 2007; Eichenbaum *et al.*, 2007). In contrast, the encoding and recognition of single items may depend more on extrahippocampal areas, such as perirhinal cortex (Vargha-Khadem *et al.*, 1997; Aggleton and Brown, 1999; Davachi, 2006; Barense *et al.*, 2007; Eichenbaum *et al.*, 2007; Murray *et al.*, 2007; Montaldi and Mayes, 2010). We sought to clarify the effects of emotion on memory by examining how it interacts with these two memory systems.

A ‘dual representation’ account proposes that emotional arousal will have different effects on these distinct memory systems (Jacobs and Nadel, 1985, 1998; Brewin *et al.*, 2010), also see (Dolcos *et al.*, 2004; Sharot *et al.*, 2004; Phelps and LeDoux, 2005; LaBar and Cabeza, 2006; Smith *et al.*, 2006; Ritchey *et al.*, 2011; Yonelinas and Ritchey, 2015) for related proposals. That is, experiencing a negative event would strengthen sensory/affective representations through amygdala up-regulation (e.g. remembering an assailant’s knife; Easterbrook, 1959; Christianson, 1992; Reisberg and Heuer, 2004). Indeed, utilising a perceptual, compared with semantic, processing strategy at encoding shows increased amygdala involvement during successful encoding and retrieval of negative images (Ritchey *et al.*, 2011; Dew *et al.*, 2014). In contrast to strengthened sensory/affective representations of items, associations between items and their context would be reduced (Mather, 2007; Touryan *et al.*, 2007; Mather and Knight, 2008; Pierce and Kensinger, 2011; Rimmele *et al.*, 2011; Madan *et al.*, 2012; Chiu *et al.*, 2013; Bisby and Burgess, 2014) due to hippocampal down-regulation (McEwen, 1999; Kim and Diamond, 2002; Becker *et al.*, 2009). Indeed, the inability to form associations between fearful stimuli and their related context is considered an important vulnerability for the development of anxiety disorders, such as post-traumatic stress disorder (PTSD; Brewin *et al.*, 2010; Acheson *et al.*, 2012).

In line with this account, a recent direct interpretation of behavioural evidence concerning emotional memory for items and associations has also suggested different roles for amygdale and hippocampi and that these structures might contribute to memory alterations in a time-dependent manner (Yonelinas and Ritchey, 2015). That is, the amygdala supports item-emotion bindings that are forgotten slowly over time due to enhanced consolidation, resulting in increased vividness and recollection ratings for emotional items (Ritchey *et al*., 2008; Sharot *et al.*, 2004; Sharot and Yonelinas, 2008). In contrast, the hippocampus supports item-context bindings, consistent with memory models of the medial temporal lobes (O’Keefe and Nadel, 1978; Cohen and Eichenbaum, 1993; Burgess *et al.*, 2002; Davachi, 2006; Diana *et al.*, 2007; Eichenbaum *et al.*, 2007), although these associations might be forgotten at a more rapid rate to accentuate item and associative memory differences (Yonelinas and Ritchey, 2015). Again, this view highlights the way in which emotion can facilitate item memory supported by the amygdala, whist associative memory processes relying on the hippocampus might be weakened or lost.

We aimed to investigate the way in which negative emotion interacts with memory by using a paired associate learning task during fMRI, in which we assessed the relationship of amygdala and hippocampal activity with memory for the items themselves and for the associations between them. Following the dual representation account, we predicted amygdala involvement in enhanced encoding of negative items. In addition, whilst we expected the hippocampus to be involved in successful associative memory, we predicted its down-regulation by the presence of negative emotion.

## Materials and methods

### Participants

Twenty (11 females) right-handed healthy volunteers with normal or corrected-to-normal vision were recruited from the University student population (mean age: 23 years, range 19–32). The study was approved by the University College London Ethics Committee and participants gave informed written consent prior to taking part in the experiment.

### Materials

A total of 300 images (150 neutral and 150 negative) were drawn from the International Affective Picture System (Lang *et al.*, 2005), Nencki Affective Picture System (Marchewka *et al.*, 2013) and our own data set. Images were chosen on the basis that they included a central person or animal and were devoid of contextual details. The majority of images used in the task included a person (four of the negative images showed an animal). We created eight different image list orders taking 200 old images and 100 new images from the overall list and these orders were counterbalanced across participants.

### Procedure

**Fig. 1:**
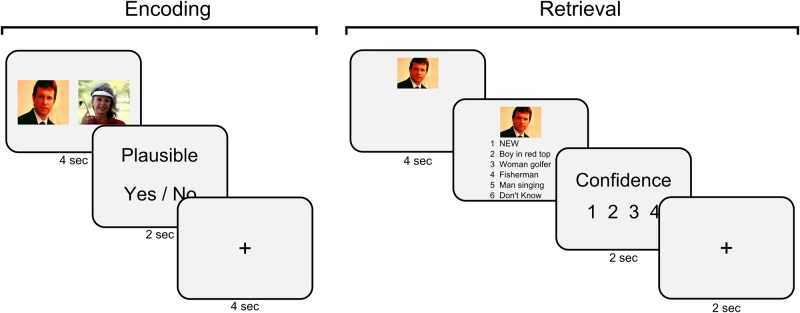
Encoding involved presentation of image pairs followed by a simple judgement of whether participants thought the two images went well together. At retrieval, participants were shown one image and instructed to try and remember the image that originally appeared with the cue image. Participants were then shown six options, including four descriptions for the possible target associate, a ‘new’ response and a ‘don’t know’ response. This was followed by a confidence rating on the participant’s response.

An example of encoding and retrieval trials is illustrated in Figure 1. At encoding, participants viewed 100 image pairs with images presented either side of each other on the screen for 4 s. Image pairs consisted of 25 pure neutral pairs, 25 pure negative pairs and 50 mixed neutral negative pairs and were presented in a randomised order. After viewing each image pair, they were given 2 s to make a simple judgment on whether they thought the two images seen went well together. This was followed by a 4 s inter-trial-interval.

After a short break (∼10 min), participants began the first retrieval phase consisting of 150 trials (100 previously seen images and 50 new images; 25 trials per emotion condition). Each test trial began with the presentation of a cue image, which could be from a previously studied pair or could be a completely novel image. During the first test session, inside the scanner, each association was only tested in one direction (e.g. cued with one image to retrieve the second image). This cue image was presented alone for 4 s and participants were instructed to remember the image that was previously shown with the cue image. After 4 s, a list of six options appeared below the cue image numbered 1–6. Four options consisted of written descriptions of images from which the participant could select the corresponding target image to go with the cue image. Two further options were labelled as ‘new’ and ‘don’t know’; to be selected if they thought the cue image was ‘new’ or if they recognised the cue as old but could not remember the target image, respectively. Selection of a response was self paced and was followed by a 1–4 confidence rating on their response (confidence therefore referred to the target associate for recognised items or for cued items given a new response). Each trial finished with a 2-s inter-trial-interval. Following completion of the scanning session, participants completed a second retrieval test outside of the scanner. This involved testing each pair in the opposite direction as tested inside the scanner (e.g. cued with the second image to retrieve the first image, reversing the cue and target valence for mixed pairs).

### fMRI acquisition

Blood oxygen level-dependent T2*-weighted functional images were acquired on a 3.0 Telsa Siemens Trio scanner using echo-planar imaging (EPI) obliquely at 45° with the following parameters: repetition time, 3360 ms; echo time, 30 ms; slice thickness, 2 mm; inter-slice gap, 1 mm; in-plane resolution, 3 × 3 mm; field of view, 64 × 72 mm^2^; 48 slices per volume. A field-map using a double echo FLASH sequence was recorded for distortion correction of the acquired EPI (Weiskopf *et al.*, 2006). After the functional scans, a T1-weighted 3D MDEFT structural image (1 mm^3^) was acquired to co-register and display the functional data. Acquisition parameters were kept constant across encoding and retrieval blocks.

### fMRI data analysis

Functional images were processed and analysed using SPM8 (www.fil.ion.ucl.uk/spm). The first five volumes from each phase were removed to allow for T1 equilibration. Standard pre-processing procedures included bias correction of EPI images to control for within-volume signal intensity differences, realignment/unwarping to correct for interscan movements, correction for differences in slice acquisition timing and normalisation of the images to an EPI template specific to our sequence and scanner that was aligned to the T1 MNI template. Finally, the normalised functional images were spatially smoothed with an isotropic 8 mm FWHM Gaussian kernel.

To assess functional data, fMRI time series were analysed using a general linear model (GLM) in which individual events were modelled based on item valence and subsequent memory. Regressors were temporally convolved with the hemodynamic response function in SPM8. Six covariates corresponding to movement parameters obtained from the realignment procedure were included in the models as regressors of no interest. The single subject parameter estimates from each session and condition taken from the first-level analysis were included in subsequent random effects analyses. For second level analyses, effects of interest were analysed using factorial analysis of variances (ANOVAs).

To assess encoding-related activity, we first examined item memory in a separate model from associative memory as some participants did not fulfil all emotional conditions for item misses (*n* = 17; three participants removed). We created separate regressors for associative hits, item hits with associative misses and complete misses across each of the three emotional conditions. Item hits with associative misses for this analysis were defined as any pair where either one of the items was correctly identified as old (in either test 1 or test 2). To assess associative memory, we examined encoding trials by creating regressors for associative memory hits (with a ‘hit’ defined as any pair for which the participant correctly retrieved the associate in either test 1 or test 2) and associative misses (associative misses were created by collapsing across item hits with associative misses and item misses) across each of the emotional conditions (pure neutral, mixed, pure negative). We collapsed across neutral-negative and negative-neutral trials at encoding as mixed pairs only differed at retrieval (depending on the cue and target valence). We then analysed the data using a 3 × 2 ANOVA (emotional condition, memory).

At retrieval, emotional conditions were split into four conditions relating to cue and target valence (pure-neutral, neutral-negative, negative-neutral, pure negative) and regressors for memory performance were created from test 1 results only (the memory test performed inside the scanner). This was to examine specific memory retrieval processes during scanning rather than a general memory effect attributed to each paired associates (i.e. taking memory results from test 1 and test 2). Similar to encoding, we assessed item memory at retrieval in a separate model, removing those participants showing zero item misses in any of the emotional conditions (*n* = 17; three participants removed). We created separate regressors for associative hits and item hits across each emotional condition and two extra regressors for neutral and negative item misses. We also included regressors for correct rejections and false alarms (collapsed across valence). Item memory hits and misses (omitting associative hits) were analysed using a 2 × 2 ANOVA (emotional condition, memory). To examine relational memory, we created a model including regressors for associative hits and associative misses (item hits with incorrect association) across the four conditions (pure neutral, neutral-negative, negative-neutral, pure negative). In addition, we included a single regressor for item misses, collapsed across the four experimental conditions due to low numbers of item misses, and additional regressors for correct rejections and false alarms (collapsed across image valence). Associative memory performance was therefore analysed using a 2 × 2 × 2 ANOVA (cue, target, memory).

### Functional connectivity analyses

Amygdalar and hippocampal activity identified in the fMRI analyses for item and associative memory were further interrogated by examining changes in functional connectivity of these structures with other brain regions between neutral and negative images. We performed a number of psychophysiological interaction (PPI; Friston *et al.*, 1997) analyses, extracting the representative time-course from voxels in the predefined region of interest (ROI; using a 6mm sphere), using the first eigenvariate calculated from singular value decomposition. The time-course from the seed region, the psychological factor (contrasting negative and neutral images), and the interaction term between the time-series and psychological factor (PPI) were then entered into a GLM.

At encoding, we performed two wholebrain PPI (Friston *et al.*, 1997) analyses. The seed region was defined as either the amygdala (ROI defined by item memory hits *vs* misses) or the hippocampus (ROI defined by associative memory hits *vs* misses). For the amygdala, we looked for increases in connectivity for negative compared with neutral images. For the hippocampus we looked for areas that showed significant increases in connectivity for neutral over negative images.

## Results

### Behavioural results

**Fig. 2:**
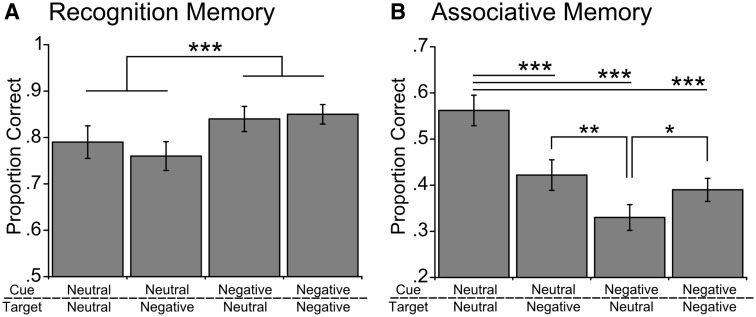
Behavioural results showing proportions correct for **(A)** item recognition and **(B)** associative memory for each type of association collapsed across tests 1 and 2. Note that mixed associations (neutral-negative and negative-neutral) switch from test 1 (testing associations in one direction inside the scanner) to test 2 testing associations in the opposite direction performed outside the scanner). See Supplementary Figure S1 for memory data split by tests 1 and 2. Bars represent SE, *P < 0.05, **P < 0.01, ***P < 0.001.

Participants completed a single encoding session and two separate memory tests. Encoding and the first memory test (test 1) were performed inside the scanner with each association tested in one direction (e.g. cue with A to retrieve B). The second memory test (test 2) was then performed during debrief outside of the scanner with all associations tested in the opposite direction from test 1 (e.g. cue with B to retrieve A; order was counterbalanced across participants). For separate test 1 and test 2 results, see supporting information Supplementary Figure S1. Behavioural results replicated the pattern found in our previous study (Bisby and Burgess 2014). Participants demonstrated enhanced recognition memory for negative cue items compared with neutral cue items [Figure 2A; *F*(1,19) = 16.02, *P *=* *0.001]. Item memory performance also decreased slightly over time from the first test session to the second test session *[*(1,19) = 6.10, *P* < 0.05; all other *P*’s > 0.17].

Cue and target valence significantly impacted associative memory performance [Figure 2B; cue × target, *F*(1,19) = 31.57, *P < *0.001; cue, *F*(1,19) = 33.11, *P *<* *0.001; target, *F*(1,19) = 3.77, *P *=* *0.07] with the presence of negative items impairing associative memory. When compared with pure-neutral associations, participants showed reduced associative memory for neutral-negative [*t*(19) = 4.76, *P* < 0.001] negative-neutral [*t*(19) = 7.88, *P* < 0.001] and pure-negative associations [*t*(19) = 5.63, *P* < 0.001]. However, associative memory was slightly boosted when the target associate was a negative item compared with a neutral item, with both neutral-negative [*t*(19) = 3.22, *P* < 0.01] and pure-negative [*t*(19) = 2.86, *P* < 0.05] associations showing better performance than negative-neutral associations (no difference between neutral-negative and negative-negative associations; *P* > 0.3). As with our item recognition results, associative memory performance showed a slight decrease over time from the first to second test session [*F*(1,19) = 5.43, *P* < 0.05; all other *P*’s > 0.47]. Importantly, observed differences across neutral and negative conditions were not driven by differences in plausibility judgments given to pairs at encoding (see Supplementary materials).

### Neuroimaging results

fMRI data were analysed at encoding and retrieval by first identifying regions where changes in activity corresponded to the valence of the presented items. We then looked for regions where changes in activity were predictive of item and associative memory. At encoding, we contrasted three valence conditions (pure neutral, pure negative, mixed), whilst at retrieval mixed pairs were further split into neutral-negative and negative-neutral depending on the valence of the presented cue item and target associate. We report family-wise error (FWE) corrected effects across the whole-brain, as well as effects in bilateral hippocampus and amygdala that survive small-volume correction (SVC) using a single bilateral hippocampal-amygdalar mask. The bilateral amygdala-hippocampal mask was created using WFU PickAtlas toolbox in SPM8, with amygdalae and hippocampi defined using the Automated Anatomical Labelling atlas. We also report effects in bilateral hippocampus and amygdala at *P* < 0.001 uncorrected threshold, treating such results with caution (see Supplementary Tables for whole brain effects at a *P* < 0.001 uncorrected threshold).

#### Encoding

Analysing subsequent item memory (emotional valence × memory; three participants removed due to few instances of item misses across all valence conditions), we first identified regions involved in viewing negative items irrespective of memory. A contrast of negative item pairs (pure-negative and mixed pairs) with pure-neutral pairs revealed activity in lateral visual regions (*P* < 0.05 FWE). No significant activity was seen in the amygdala at a liberal threshold (*P* < 0.001 uncorrected).

**Fig. 3:**
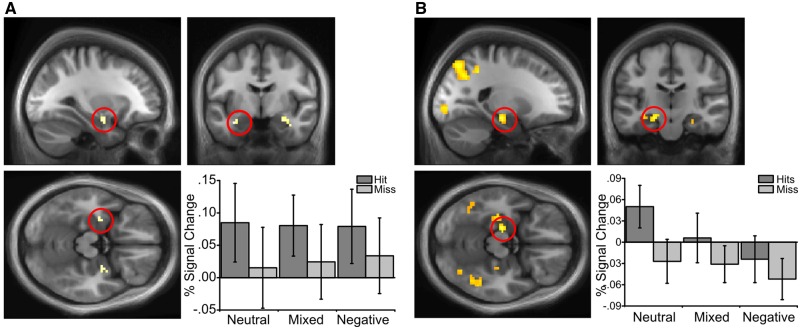
Encoding activity showing subsequent memory effects across emotional valence conditions. **(A)** A contrast of subsequent item hits *vs* misses identified the left amygdala (*P* < * *0.001 uncorrected; −33, −6, −21). **(B)** Activations in the left anterior hippocampus predicted subsequent associative hits compared with misses (*P* < 0.05 FWE SVC; −21, −18, −15). Effects displayed on group average brains and thresholded at *P* < 0.005 uncorrected for display purposes.

Comparing item hits with misses revealed a subsequent memory effect in the left amygdala (*P* < 0.001 uncorrected; Figure 3A; note that this amygdala effect did not survive SVC). No significant activity was seen in the hippocampus for this contrast at an uncorrected threshold (*P* < 0.001 uncorrected). We also saw regions that exhibited a significant interaction between item valence and subsequent item memory, including an area in the right lateral occipital cortex (*P* < 0.05 FWE), which showed a greater subsequent memory effect (item hits *vs* misses) for pairs that included a negative item *vs* pure neutral pairs. We also note that a direct comparison between pure neutral and pure negative conditions (omitting mixed valence trials) showed an interaction in an area of the left perirhinal cortex with a greater hits *vs* misses effect for pure negative compared with pure neutral trials using a lower threshold (*P* < 0.005 uncorrected). However, we do not draw strong conclusions from this finding given as it was only seen at a reduced threshold. Thus, whilst the amygdala predicted subsequent item memory (at an uncorrected threshold) regardless of valence, the lateral occipital cortex predicted subsequent item memory selectively for pairs that included a negative item.

Comparing associative hits *vs* misses revealed a subsequent associative memory effect in the left anterior hippocampus (*P* < 0.05 FWE SVC; Figure 3B) irrespective of valence. However, there was also a main effect of valence within a similar hippocampal region. A two-way ANOVA (emotional valence × memory) on activity in this hippocampal ROI yielded significant main effects of emotional valence [*F*(2,38) = 3.65, *P* < 0.05] and subsequent associative memory [*F*(1,19) = 18.96, *P* < 0.001; as expected given this ROI was defined by this contrast], but no significant interaction (*F* < 1). Thus activity in anterior hippocampus during encoding was increased for subsequently remembered associations and was also reduced by the presence of negative images. No subsequent associative memory effect was seen in the amygdala even at a liberal *P* < 0.001 uncorrected threshold.

#### Retrieval

**Fig. 4:**
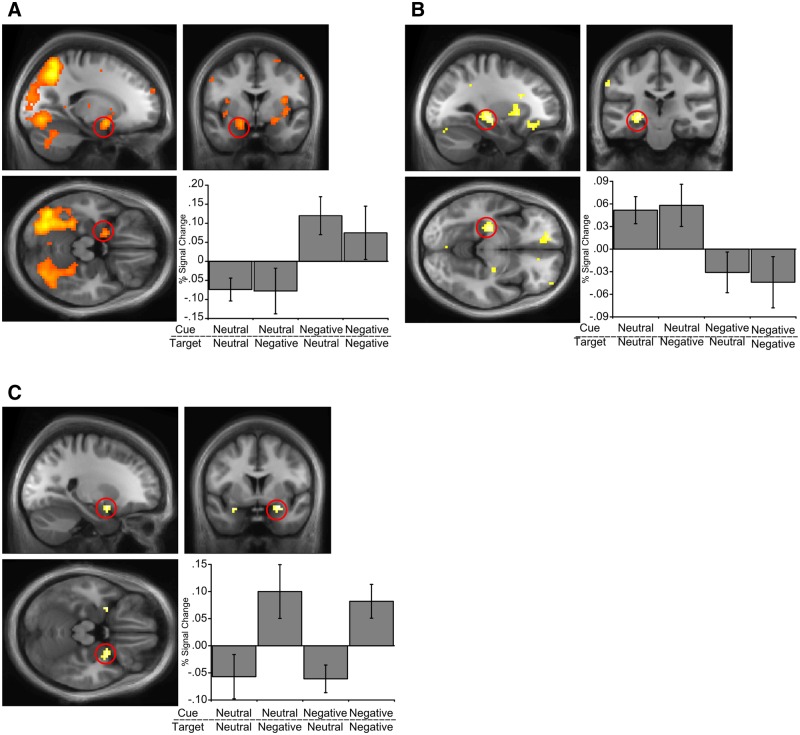
Retrieval activity related to item and associative memory (percent signal change figures show associative hits minus misses for each condition) across cue and target conditions. **(A)** Activity in the amygdala was increased for negative item memory (hits minus misses) compared with neutral item memory (*P* < 0.05 FWE SVC; −18, 0, −15). **(B)** We saw a significant activation in the left hippocampus (*P* = 0.07 FWE SVC; −27, −24, −9) for associative memory (associative hits minus misses) when cued with a neutral item *vs* a negative item. **(C)** Activity in the right amygdala increased during successful associative memory (associative hits minus misses) when retrieving a negative target associate compared with a neutral target (*P* < 0.05 FWE SVC; +30, +3, −15). Effects displayed on group average brains and thresholded at *P* < 0.005 uncorrected for display purposes.

Examining item memory across emotional valence conditions at retrieval (item hits without associative hits *vs* item misses; similar to encoding, three participants were removed due to low numbers of item misses in some valence conditions), we saw an item valence effect irrespective of memory success in the amygdala (*P* < 0.05 FWE SVC; −21, −3, −22), and lateral occipital cortex (similar to encoding; *P* < 0.05 FWE) when viewing negative compared with neutral items. There was a significant interaction between item memory and valence in bilateral amygdala, reflecting a memory effect for negative items (hits *vs* misses) but not neutral items (*P* < 0.05 FWE SVC; −18, 0, −15; 24, 0, −12; Figure 4A). Thus, activity in the amygdala correlated with both viewing negative items and recognising negative items.

Associative memory at retrieval was analysed using a 2 × 2 × 2 ANOVA (cue valence, target valence, associative hits *vs* misses), revealing a significant interaction between cue valence and memory in the left hippocampus (*P* = 0.07 FWE SVC; *P* < 0.001 uncorrected; −27, −24, −9; Figure 4B) reflecting an associative memory effect when cued with a neutral, but not a negative item, irrespective of the valence of the target item. Thus, the presence of a negative cue item disrupts the association between hippocampal activity and relational retrieval (though we note this effect did not survive SVC).

We also saw a target valence × memory interaction in the right amygdala (*P* < 0.05 FWE SVC; +30, +3, −15; Figure 4C), reflecting a significant associative memory effect (associative hits > misses) when retrieving a negative, but not a neutral target (irrespective of the cue valence). Amygdala activity therefore correlates with relational memory retrieval success, but only when retrieving a negative item from memory. That is, when the associated item is successfully retrieved, amygdala activity correlates with the valence of that item.

#### Functional connectivity

We assessed how the amygdala might influence item memory encoding via functional connectivity with other regions. For example, previous work has suggested that the amygdala interacts with visual areas to promote attention and encoding of emotionally relevant stimuli (Vuilleumier *et al.*, 2004). We therefore performed a wholebrain PPI analysis with the left amygdala as a seed region, taken from the encoding subsequent item memory effect, and looked for areas showing greater connectivity for negative over neutral images. This analysis revealed significant connectivity between amygdala and lateral occipital cortex (*P* < 0.001 uncorrected; +39, −63, +3; z-score = 4.38), supporting the view that the amygdala interacts with visual areas during encoding of negative stimuli.

We next performed a wholebrain PPI analysis to examine functional connectivity between the hippocampus and other brain regions that differs for encoding of neutral and negative images. Taking the hippocampal location showing an associative subsequent memory effect as a seed region, we looked for regions showing greater connectivity for neutral over negative images. We saw significant functional connectivity with the left entorhinal cortex for neutral images (*P* < 0.001 uncorrected; −12, −9, −30; z-score = 3.98), suggesting that whilst the hippocampus works in concert with other medial temporal lobe structures for successful encoding, this coupling is reduced during the presentation of negative items.

## Discussion

We investigated how emotion affects memory formation and retrieval by examining the effects of negative emotion on item and associative memory. Our results are in line with a ‘dual-representation’ account that predicts differential effects of negative emotion on amygdalar- and hippocampal-dependent systems, resulting in item memory enhancements and associative memory impairments. We observed a clear dissociation between the effects of negative emotion on the neural activity related to item and associative memory. Whereas the amygdala was ‘*up-regulated*’ by negative emotion consistent with the enhanced encoding and retrieval of negative items themselves, the hippocampus was ‘*down-regulated’* by the presence of negative items with a corresponding decrease in associative memory performance.

### Amygdala up-regulation and item memory

Up-regulation of the amygdala and visual regions by negative items may reflect enhanced perception of, and attention to, these stimuli (Vuilleumier *et al.*, 2001, 2004; Phelps and LeDoux, 2005). This would contribute to enhanced recognition memory for these items, consistent with previous accounts of emotional memory. Although greater activity in visual regions predicted subsequent memory for negative items, areas often associated with high-level visual recognition (Kanwisher, 2010), the amygdala item memory effect at encoding was seen irrespective of valence, suggesting that it plays a more general role in item memory encoding (Fried *et al.*, 1997; Rutishauser *et al.*, 2010; Farovik *et al.*, 2011; Kaplan *et al.*, 2014). However, we did see greater functional connectivity between the amygdala and visual areas for negative images, supporting the view that the amygdala might specifically enhance item memory for negative stimuli through communication with visual regions to facilitate perception and attention that would lead to enhanced encoding (Vuilleumier *et al.*, 2004). We also note that it is plausible that our study was underpowered to detect item recognition effects in the amygdala specific to negative stimuli given that previous studies showing such effects often use a high number of negative stimuli (e.g. Kensinger and Schacter, 2006). However, the amygdala may play a more specific role in the consolidation of negative compared with neutral items that would occur following encoding (McGaugh, 2000, 2004; Talmi, 2013; Yonelinas and Ritchey, 2015).

Observed increases in amygdalar activity at test also suggests its role in item memory retrieval (Dolcos *et al.*, 2004; Sharot *et al.*, 2004; Smith *et al.*, 2004; Dew *et al.*, 2014). There was greater amygdala activity for recognised negative items than forgotten ones. The amygdala may therefore play a role in retrieving negative affective value from a previous experience, in line with fear conditioning accounts (Ledoux, 2000; Maren and Quirk, 2004; Fanselow and Poulos, 2005). This interpretation was further supported by activity patterns during associative memory retrieval. Irrespective of whether cued with a neutral or negative item, amygdala activity increased with successful retrieval of a negative target associate.

As our results show, reinstatement of the affective properties of a memory not only occurred when a negative item was recognised, but also when a negative item was successfully retrieved through relational memory processes. The amygdala might function by attaching affective/reinforcement properties to items, possibly through its connections with extrahippocampal area such as perirhinal cortex (Paz *et al.*, 2006; Farovik *et al.*, 2011). In line with this view, we did see a greater, albeit weak, item memory effect in perirhinal cortex during encoding of negative compared with neutral items. This would provide a potential mechanism by which memory for items would be enhanced. Reinstating the affective components linked to an item in memory could also explain its contribution to increases in vividness and confidence often reported for negative memories (Neisser and Harsch, 1992; Sharot *et al.*, 2004).

### Hippocampal down-regulation and associative memory

The literature is unclear on how negative emotion is expected to interact with hippocampal function and corresponding associative memory. However, a dual representation account predicts that the experience of negative emotion will down-regulate hippocampal function. We provide evidence of hippocampal down-regulation during the presence of negative items at encoding. This down-regulation corresponded to a behavioural reduction in associative memory for pairs including a negative item (Kensinger and Schacter, 2006; Mather *et al.*, 2006; Touryan *et al.*, 2007; Rimmele *et al.*, 2011). The exact mechanisms underpinning hippocampal down-regulation are unclear. Interestingly, we did not see an inverse relationship between hippocampal and amygdalar activity during encoding suggesting that the two structures do not necessarily work in direct opposition to affect memory.

It is proposed that interactions between slow elevations in cortisol and rapid arousal-induced noradrenergic activation might work in concert to promote more lower-level processing, whilst impairing higher level memory processes and hippocampal function (Jacobs and Nadel, 1985, 1998; McEwen, 1999; Kim and Diamond, 2002; Becker *et al.*, 2009; Joëls *et al.*, 2011; van Ast *et al.*, 2013). Although the intensity of our negative images might not reach the stress response often reported following acute cortisol administration (images would also have been encoded as cortisol increased rather than at peak), it is possible that the combination of highly arousing negative images and a task that places large associative memory demands on the hippocampal system increases sensitivity to relatively mild levels of stress. Whether changes in hippocampal function during negative events, as observed in our study, reflect a specific disruption or a ‘shift’ in resources to structures involved in promoting lower level perceptual processing remains unclear.

During encoding, we saw increased hippocampal activity for paired associations that were subsequently remembered, and that hippocampal activity was reduced by the presence of a negative item. Further, our encoding results also revealed greater functional connectivity between the hippocampus and entorhinal cortex for neutral compared with negative images. During retrieval, we saw increased hippocampal activity for successful associative retrieval, but only when cued by a neutral item. Our results support the established role of the hippocampus in associative memory representations and its interaction with extrahippocmpal structures to support memory (O’Keefe and Nadel, 1978; Cohen and Eichenbaum, 1993; Vargha-Khadem *et al.*, 1997; Aggleton and Brown, 1999; Burgess *et al.*, 2002; Davachi, 2006; Barense *et al.*, 2007; Eichenbaum *et al.*, 2007; Murray *et al.*, 2007; Montaldi and Mayes, 2010). Weakened associative memory for negative items might result from both down-regulations of encoding-related hippocampal activity, and by disruption of retrieval-related hippocampal activity if the retrieval cue is negative. In addition, this down-regulation might disrupt efficient communication with other key medial temporal lobe memory-related structures. These results are consistent with previous studies demonstrating stress-induced impairments in hippocampal-dependent retrieval (Roozendaal *et al.*, 2002; de Quervain *et al.*, 2009).

Amygdala responses during successful associative retrieval (associative hits *vs* misses) corresponded to a similar pattern of associative memory performance with greater amygdala activity when successfully retrieving a negative compared with neutral target associate. Although the presence of a negative item impaired associative memory performance compared with neutral–neutral pairs, for pairs including a negative item retrieval of a negative target associate was better than retrieval of a neutral target, see also (Madan *et al.*, 2012; Bisby and Burgess, 2014). We suggest that this memory facilitation effect is, in part, due to enhancement OF amygdala-dependent? item-memory, given the increased amygdala activity for successful retrieval of a negative target compared with a neutral target. That is, associations between items and their affective response, mediated by the amygdala, might enhance their retrievability from memory, even in conditions when hippocampal-dependent associative memory representations would be weakened.

We are the first to use neuroimaging to examine the effect of negative emotion on paired associate learning in this way, whilst similar designs have been used to assess the associative relationship between items for neutral events (Kahana, 2002). By using an associative memory paradigm that presented participants with written descriptions to select the correct paired associates, we were able to remove confounding effects of showing negative retrieval targets at test. Participants were therefore instructed to try and retrieve the paired associate following cue presentation and then select the description that best described the image they had retrieved, potentially relying more on recollective memory processes. This approach differs from previous reports that ask participants an animacy or commonness question during the encoding trial (Kensinger and Schacter, 2006) or required them to retrieve the affective component of a paired associate but not the specific details of the image (Smith *et al.*, 2004, 2006). Such studies have proposed that retrieval of a negative associate correlates with increased connectivity between the hippocampus and amygdala (Smith *et al.*, 2006). This suggests that, if increased amygdala activity enhances retrieval of negative targets, it does so in concert with the hippocampus, despite the overall reduction in hippocampal activity.

Our findings have important implications for the understanding of psychological disorders that involve emotional memory disturbances, such as PTSD. A primary feature of PTSD is the repeated occurrence of distressing imagery in response to a trauma that involuntarily enters consciousness. Dual representation accounts (Jacobs and Nadel, 1985, 1998; Brewin *et al.*, 2010) suggest that reduced hippocampal function in the presence of negative stimuli could impair the formation of important associations between items and their context. When combined with strengthened sensory/perceptual images of key features from the event (e.g. the oncoming headlights from a car before an accident; Ehlers and Clark, 2000), these strong sensory laden images might be triggered out of context in the form of intrusive memories. Exposure therapy (Foa and Rothbaum, 1998) and imagery re-scripting techniques (Hackmann, 1998) aim to strengthen associations between negative intrusive imagery and contextual information to abolish unwanted intrusive imagery. Our findings help to understand the basic mechanisms that might underpin the effects of emotion on memory for sensory stimuli and their associated contexts, and thus help to understand how these techniques could work and how they might be strengthened and refined.

## Conclusions

We provide support for a dual representation account of emotional memory, with dissociable roles for the amygdala and hippocampus in memory function and opposing effects of negative affect upon these roles. Our results suggest that down-regulation of hippocampal activity by negative affect specifically reduces associative memory performance, leaving item memory unimpaired, whereas up-regulation of amygdala activity by negative affect boosts memory for the negative item itself. These findings have important implications for the development and treatment of negative symptoms in anxiety disorders, such as PTSD.

## Supplementary Material

Supplementary Data
