# Supplementary material for: Opposing effects of negative emotion on amygdalar and hippocampal memory for items and associations
Source: Soc Cogn Affect Neurosci. 2016 Mar 12;11(6):981–90. doi: 10.1093/scan/nsw028 (PMC4884322; doi:10.1093/scan/nsw028)
Supplement: Supplementary Data [file supp_11_6_981__index.html]

Opposing effects of negative emotion on amygdalar and hippocampal memory for items and associations — Opposing effects of negative emotion on amygdalar and hippocampal memory for items and associations — Supplementary Data 

# Opposing effects of negative emotion on amygdalar and hippocampal memory for items and associations

## Supplementary Data

files

- Supplementary Data - docx file
