# Supplementary material for: Opposing effects of negative emotion on amygdalar and hippocampal memory for items and associations
Source: Soc Cogn Affect Neurosci. 2016 Mar 12;11(6):981–90. doi: 10.1093/scan/nsw028 (PMC4884322; doi:10.1093/scan/nsw028)
Supplement: Supplementary Data [file supp_nsw028_scan-15-404-File006.docx]

**Supplementary information**


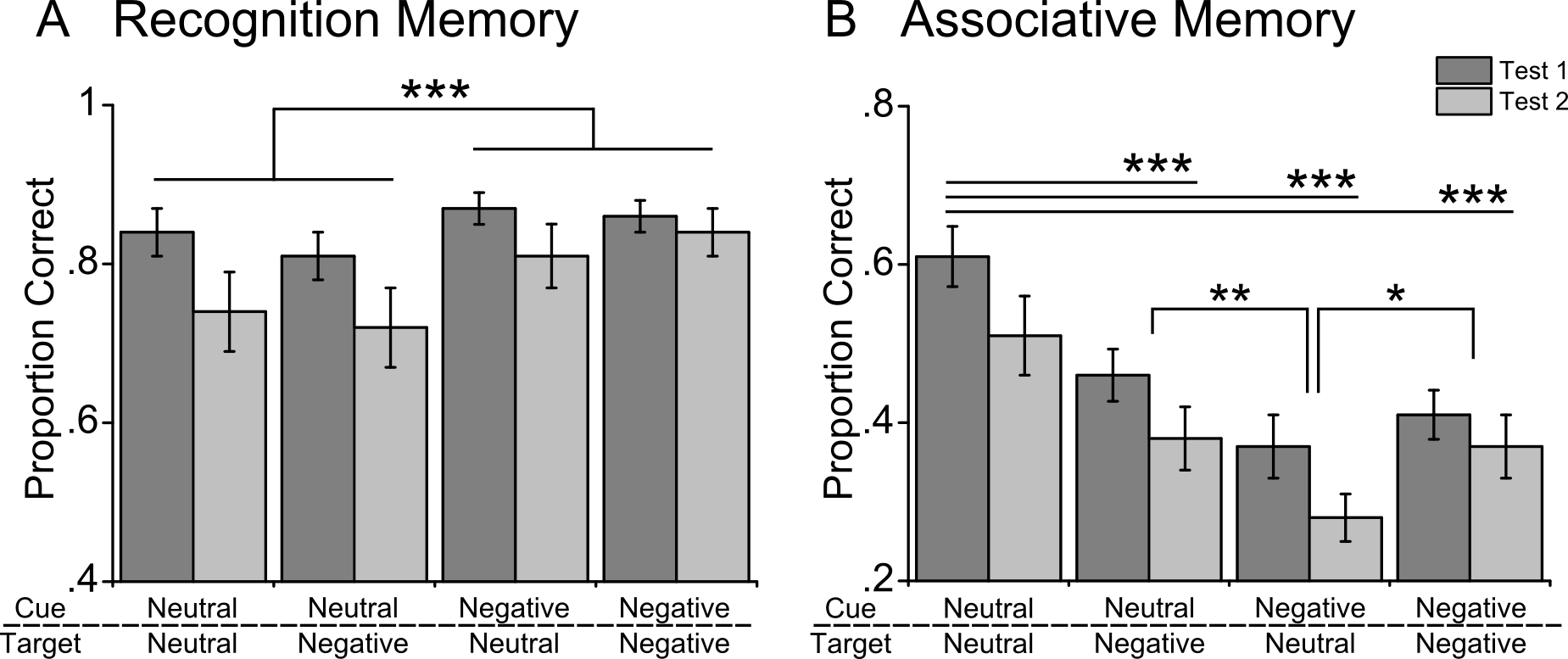


**Figure S1.** Behavioural results showing proportions correct for (A) recognition and (B) associative memory performance for each type of association across test 1 and test 2 separately. Bars represent standard error, *p<0.05, **p<0.01, ***p<0.001.

**Memory performance and plausibility ratings**

As participants were asked to make a plausibility rating for each paired associate at encoding, we performed further analyses on these plausibility judgments to rule out whether differences in responses across conditions might explain our results. First, we performed a 3 x 2 ANOVA on the proportion of yes and no plausibility responses across the three conditions (pure neutral, mixed, pure negative) at encoding, irrespective of memory performance. We found no differences between response type across any of the conditions (condition x response, F(2,38)=0.62, p=0.54; condition, F(2,38)=2.37, p=0.11; response type, F(1,19)=0.06, p=0.82). In addition, we assessed whether differences in plausibility responses could explain the pattern of associative memory performance. We therefore carried out a 2 x 2 x 2 ANOVA (cue, target, response type) on associative memory performance. As reported in our main results, analysis showed a cue x target interaction (F(1,19)=8.24, p = 0.01), a main effects of target (F(1,19)=24.15, p<0.001) and main effect of cue that approached significance (F(1,19)=3.16, p=0.09). Importantly, plausibility responses did not interact with we any of our conditions of interest (cue x target x response, F(1,19)=1.08, p=0.31; cue x response, F(1,19)=1.71, p=0.21; target x response, F(1,19)=0.27, p=0.62), nor did we see a main effect of response type (F(1,19)=0.08, p=0.79). In summary, these results suggest that the pattern of our behavioural data was not driven by differences in plausibility responses given to neutral and negative paired associates at encoding.

| **Table S1. Mean (SD) proportion of plausibility judgments across conditions.** | | |
| --- | --- | --- |
|  | Plausibility Judgment | |
|  | Yes | No |
| **Proportion of responses split by condition** |  |  |
| Pure Neutral | 0.52 ± 0.26 | 0.44 ± 0.25 |
| Mixed | 0.45 ± 0.30 | 0.51 ± 0.30 |
| Pure Negative | 0.49 ± 0.26 | 0.45 ± 0.26 |
|  |  |  |
| **Proportion of associative hits split by plausibility responses** |  |  |
| Pure Neutral | 0.34 ± 0.25 | 0.27 ± 0.20 |
| Neutral-Negative | 0.20 ± 0.16 | 0.26 ± 0.18 |
| Negative-Neutral | 0.20 ± 0.19 | 0.18 ± 0.12 |
| Pure-Negative | 0.22 ± 0.17 | 0.19 ± 0.13 |
|  |  |  |

| **Table S2. Summary of imaging findings for valence and item memory during encoding.** | | | | | | | |
| --- | --- | --- | --- | --- | --- | --- | --- |
|  |  | MNI coordinates | | | | |  |
| Region | Laterality | x |  | y |  | z | z-score |
| **Emotional valence x item memory** | | | | | | | |
| **Item valence effect (negative > pure-neutral)** | | | | | | | |
| Inferior temporal gyrus | R | 45 |  | -60 |  | -6 | 6.85 |
|  | L | -48 |  | -66 |  | -9 | 6.43 |
| Inferior occipital gyrus | L | -30 |  | -84 |  | 12 | 6.63 |
| Middle occipital gyrus | R | 36 |  | -78 |  | 9 | 6.19 |
| Lingual gyrus | R | 21 |  | -81 |  | -9 | 4.70 |
| SupraMarginal gyrus* | L | -60 |  | -24 |  | 36 | 4.23 |
| Postcentral gyrus* | R | 63 |  | -18 |  | 33 | 3.35 |
|  |  |  |  |  |  |  |  |
| **Main effect item memory (hits > misses)** | | | | | | | |
| Inferior temporal gyrus* | R | 48 |  | -60 |  | -6 | 4.23 |
|  | L | -45 |  | -57 |  | -12 | 3.28 |
| Amygdala * | L | -33 |  | -3 |  | -18 | 3.39 |
|  |  |  |  |  |  |  |  |
| **Emotional valence(negative > neutral) x item memory (hits > misses) interaction** | | | | | | | |
| Inferior temporal gyrus | R | 42 |  | -63 |  | -6 | 4.50 |
| Inferior temporal gyrus * | L | -48 |  | -63 |  | -6 | 3.76 |
|  |  |  |  |  |  |  |  |
| **Emotional valence(neutral > negative) x item memory (hits > misses) interaction** | | | | | | | |
| Inferior parietal lobule | R | 51 |  | -51 |  | 45 | 4.83 |
| Inferior parietal lobule* | L | -42 |  | -54 |  | 39 | 4.53 |
| Superior frontal gyrus* | R | 27 |  | 54 |  | 15 | 4.20 |
| Middle orbital gyrus* | L | -36 |  | 57 |  | -6 | 4.00 |
| Middle cingulate cortex * | R | 3 |  | -24 |  | 39 | 4.10 |
| Middle temporal gyrus* | R | 54 |  | -36 |  | 3 | 3.90 |
|  | L | -66 |  | -36 |  | -3 | 3.75 |
| Precuneus* | R | 12 |  | -69 |  | 30 | 4.07 |
|  |  |  |  |  |  |  |  |
| p<0.05 FWE across whole brain unless stated; ^┼^p<0.05 FWE SVC; *p<0.001 uncorrected | | | | | | | |

| **Table S3. Summary of imaging findings for associative memory during encoding.** | | | | | | | |
| --- | --- | --- | --- | --- | --- | --- | --- |
|  |  | MNI coordinates | | | | |  |
| Region | Laterality | x |  | y |  | z | z-score |
| **Emotional valence x associative memory** | | | | | | | |
| **Main effect of memory (hits > misses)** | | | | | | | |
| Middle occipital gyrus | R | 33 |  | -72 |  | 33 | 4.84 |
|  | L | -24 |  | -66 |  | 36 | 3.92 |
| Hippocampus^┼^ | L | -21 |  | -18 |  | -15 | 3.80 |
| Inferior frontal gyrus * | L | -48 |  | 30 |  | 18 | 4.53 |
|  | R | 51 |  | 33 |  | 15 | 3.51 |
| Inferior occipital gyrus * | L | -30 |  | -84 |  | -6 | 4.04 |
| Middle temporal gyrus * | R | 51 |  | -57 |  | -3 | 4.03 |
| Inferior parietal lobule* | L | -48 |  | -42 |  | 54 | 3.13 |
|  |  |  |  |  |  |  |  |
| p<0.05 FWE across whole brain unless stated; ^┼^p<0.05 FWE SVC; *p<0.001 uncorrected | | | | | | | |

| **Table S4. Summary of imaging findings for valence and item memory during retrieval.** | | | | | | | |
| --- | --- | --- | --- | --- | --- | --- | --- |
|  |  | MNI coordinates | | | | |  |
| Region | Laterality | x |  | y |  | z | z-score |
| **Emotional valence x item memory** | | | | | | | |
| **Negative cues > neutral cues** | | | | | | | |
| Middle occipital gyrus | L | -33 |  | -84 |  | 12 | >8.00 |
|  | R | 36 |  | -78 |  | 15 | >8.00 |
| Inferior temporal gyrus | R | 48 |  | -63 |  | -6 | >8.00 |
| Superior parietal lobule | L | -18 |  | -66 |  | 45 | 6.75 |
| Amygdala^┼^ | L | -21 |  | 3 |  | -21 | 3.69 |
| Middle frontal gyrus* | R | 48 |  | 42 |  | 9 | 4.14 |
| Superior frontal gyrus* | L | -27 |  | 3 |  | -27 | 3.94 |
| SupraMarginal gyrus* | L | -60 |  | -24 |  | 33 | 3.66 |
|  | R | 60 |  | -21 |  | 30 | 3.55 |
|  |  |  |  |  |  |  |  |
| **Main effect of item memory (hits > misses)** | | | | | | | |
| Inferior frontal gyrus | L | -45 |  | 12 |  | 27 | 7.45 |
| Middle temporal gyrus | L | -57 |  | -39 |  | 3 | 5.43 |
| Inferior frontal gyrus | L | -39 |  | 33 |  | -12 | 5.21 |
| Superior parietal lobule | L | -30 |  | -60 |  | 45 | 5.16 |
| Inferior occipital gyrus* | R | -27 |  | -87 |  | -6 | 4.17 |
| Middle occipital gyrus* | L | 33 |  | -87 |  | 33 | 4.14 |
|  | | | | | | | |
| **Emotional valence (negative > neutral) x item memory (hits > misses) interaction** | | | | | | | |
| Middle occipital gyrus | L | -33 |  | -84 |  | 15 | 7.20 |
|  | R | 36 |  | -81 |  | 6 | 6.27 |
| Inferior temporal gyrus | R | 48 |  | -60 |  | -9 | 6.60 |
| Amygdala^┼^ | R | -18 |  | 0 |  | -15 | 4.37 |
|  | L | 24 |  | 0 |  | -12 | 3.62 |
| Inferior frontal gyrus* | R | 51 |  | 42 |  | 6 | 4.67 |
| Precentral gyrus* | R | 51 |  | 6 |  | 30 | 4.10 |
| Superior frontal gyrus* | L | -21 |  | 60 |  | 27 | 4.01 |
|  | R | 24 |  | 54 |  | 36 | 3.84 |
| Middle frontal gyrus* | R | 36 |  | 30 |  | 48 | 3.70 |
|  |  |  |  |  |  |  |  |
| p<0.05 FWE across whole brain unless stated; ^┼^p<0.05 FWE SVC; *p<0.001 uncorrected | | | | | | | |

| **Table S5. Summary of imaging findings for associative memory during retrieval.** | | | | | | | |
| --- | --- | --- | --- | --- | --- | --- | --- |
|  |  | MNI coordinates | | | | |  |
| Region | Laterality | x |  | y |  | z | z |
| **Emotional valence x associative memory** | | | | | | | |
| **Main effect of memory (hits > misses)** | | | | | | | |
|  |  | -3 |  | -27 |  | 0 | 5.92 |
| Inferior frontal gyrus | L | -48 |  | 9 |  | 24 | 5.64 |
| Caudate nucleus | R | 9 |  | 12 |  | 3 | 5.55 |
|  | L | -9 |  | 12 |  | -3 | 5.31 |
| Middle occipital gyrus | L | -30 |  | -69 |  | 39 | 5.36 |
| Thalamus | L | -6 |  | -12 |  | 12 | 4.95 |
| Inferior parietal lobule | L | -36 |  | -45 |  | 39 | 4.93 |
| Middle temporal gyrus* | L | -57 |  | -48 |  | -6 | 4.57 |
| Middle frontal gyrus* | R | 39 |  | -6 |  | 57 | 3.80 |
|  |  |  |  |  |  |  |  |
| **Cue (neutral > negative) x memory (hits > misses) interaction** | | | | | | | |
| Hippocampus* | L | -27 |  | -24 |  | -9 | 3.39 |
|  |  |  |  |  |  |  |  |
| **Target (negative > neutral) x memory (hits > misses) interaction** | | | | | | | |
| Amygdala^┼^ | R | 27 |  | 3 |  | -15 | 3.63 |
| Amygdala* | L | -30 |  | 3 |  | -18 | 3.32 |
| Postcentral gyrus* | L | -66 |  | -21 |  | 24 | 3.73 |
|  |  |  |  |  |  |  |  |
| p<0.05 FWE across whole brain unless stated; ^┼^p<0.05 FWE SVC; *p<0.001 uncorrected | | | | | | | |
